# Supplementary material for: The interactome of intact mitochondria by cross-linking mass spectrometry provides evidence for coexisting respiratory supercomplexes
Source: Mol Cell Proteomics. 2017 Dec 8;17(2):216–32. doi: 10.1074/mcp.RA117.000470 (PMC5795388; doi:10.1074/mcp.RA117.000470)
Supplement: Supplemental Data [file supp_RA117.000470_133922_0_supp_23978_fzffwf.zip › spectra_annotation/mito_DR_spectra_annotation/249-1-4-1-12-1.pdf]

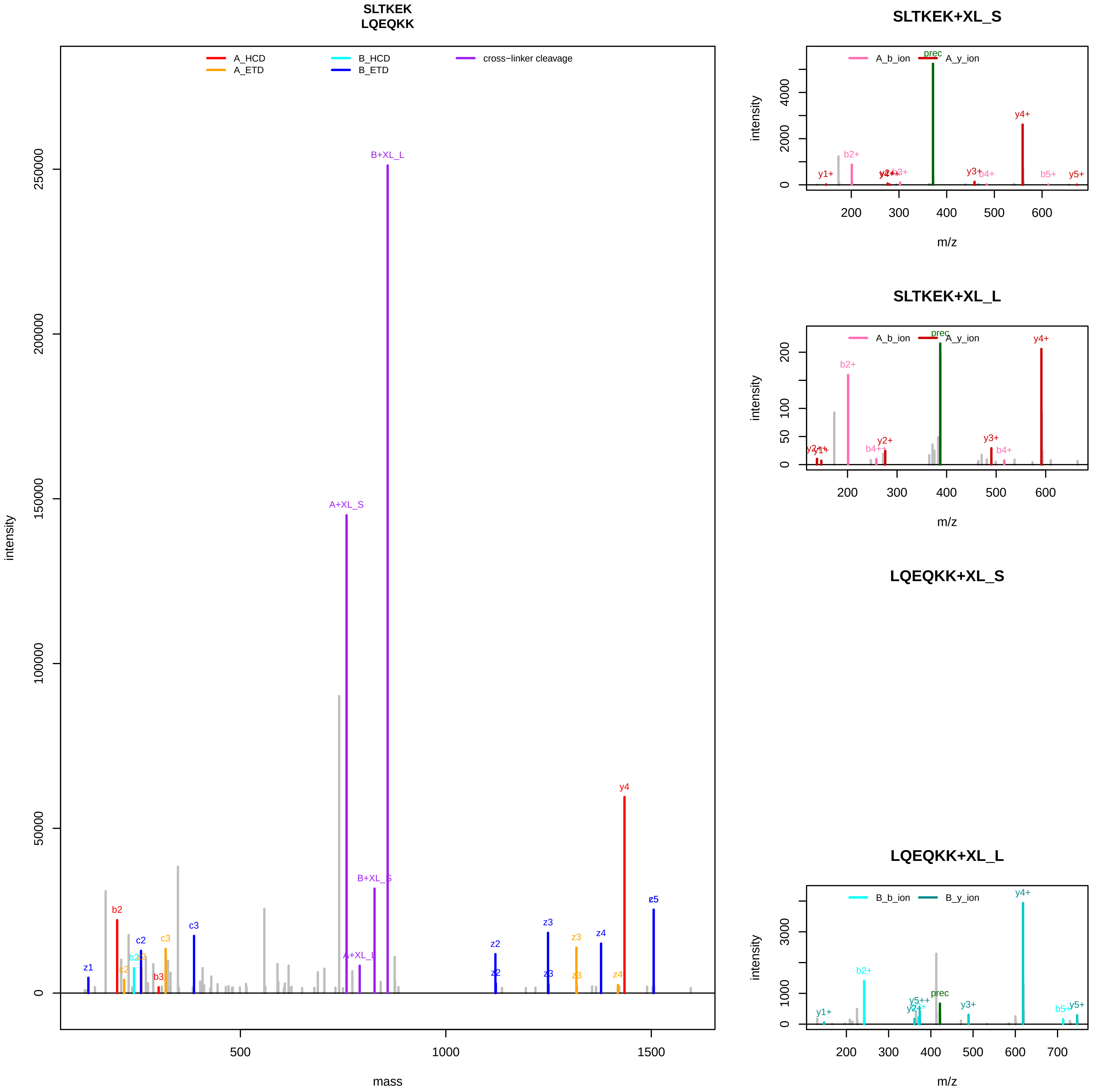
SLTKEK
LQEQKK
A_HCD
A_ETD
B_HCD
B_ETD
cross−linker cleavage
B+XL_L
A+XL_S
B+XL_S
A+XL_L
y4
z5
z1
b2
c2
b2
c3
b3
z2
z3
z4
intensity
mass
0
50000
100000
150000
200000
250000
500
1000
1500
SLTKEK+XL_S
A_b_ion
A_y_ion
prec
b2+
y1+
y4+
y3+
b4+
b5+
y5+
m/z
SLTKEK+XL_L
y2+
LQEQKK+XL_S
LQEQKK+XL_L
B_b_ion
B_y_ion
b5+
